# *FOXO3* cell resilience gene neighborhood

**DOI:** 10.18632/aging.101349

**Published:** 2017-12-14

**Authors:** Timothy A. Donlon, Bradley J. Willcox, Brian J. Morris

**Affiliations:** Basic & Clinical Genomics Laboratory, School of Medical Sciences and Bosch Institute, University of Sydney, New South Wales, Australia

**Keywords:** FOXO3, gene-gene interactions, healthy aging, longevity, fluorescent in situ hybridization

The forkhead/winged helix box gene, group O, type 3 (Foxo3) transcription factor regulates a wide array of processes that confer cell resilience and healthy aging. The FoxO3 gene (*FOXO3*) is a well-established longevity gene in which favourable alleles confer protection against mortality from coronary heart disease [1]. A recent study reported gene-gene interactions between *FOXO3* and 46 flanking genes on chromosome 6q21 [2]. *FOXO3* was located at the hub of this early-replicating, highly conserved region of these co-expressed genes. Like *FOXO3*, the 46 neighboring genes are involved in various processes that contribute to cell resilience, namely autophagy, stress response, energy/nutrient sensing, cell proliferation, apoptosis and stem cell maintenance. Together they may work as a “gene factory” for healthy aging.

The interaction of *FOXO3* with its neighboring genes involved CCCTC-binding factor zinc finger protein (CTCF), a transcription factor that regulated chromatin architecture, attracting tissue-specific transcriptional activators, repressors, cohesin and RNA polymerase II. CTCF makes contact with tens of thousands of sites across the genome by using different combinations of its 11 zinc finger domains to bind different DNA target sequences and proteins. CTCF brings *cis*-regulatory elements together into co-regulated islands several hundred kb in size. Multiple islands are then brought together into a functional neighborhood, or “archipelago”, of 3–5 Mb. The action of CTCF causes the looping of chromatin between the CTCF binding sites on DNA. It was found that in response to stress, *FOXO3* moved towards its neighboring genes, as visualized by fluorescent in situ hybridization (FISH) of lymphoblastoid cell lines (Figure 1, top). Measurements in the FISH experiments further showed that the clustering of *FOXO3* with its neighboring genes following stress was greater for carriers of the longevity-associated *G*-allele of *FOXO3* SNP *rs2802292* compared with the common genotype *TT*. *FOXO3* mRNA expression in response to stress was more pronounced for *FOXO3 G*-allele carriers (Figure 1, bottom). *HACE1* and *AMD1* were also activated by stress. The response of all 46 genes has not yet been tested.

**Figure 1:**
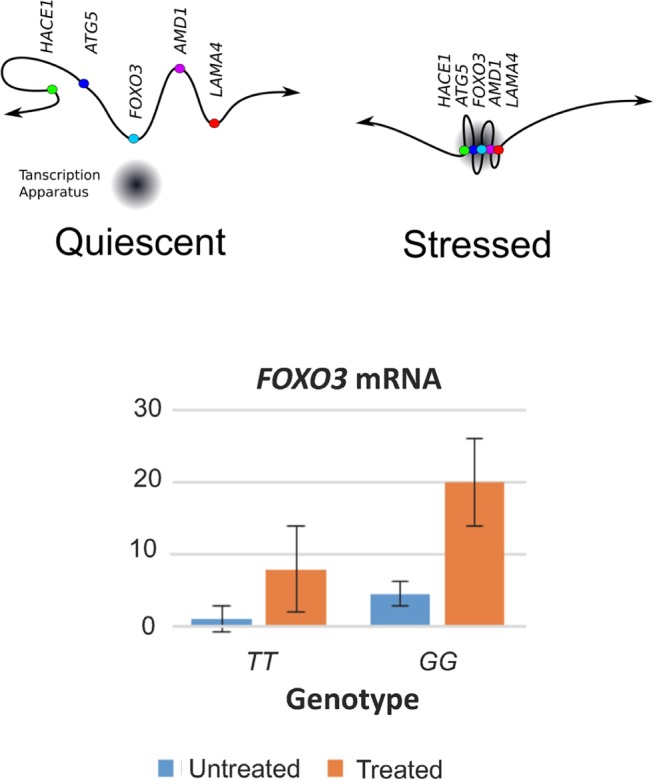
Dynamics of *FOXO3* in lymphoblastoid cell lines *Top:* Schematic depicting results of separate FISH experiments (*HACE1-FOXO3-LAMA4* and *ATG5-FOXO3-AMD1*) in which, in response to stress (200 μM H_2_O_2_ and serum deprivation), *FOXO3* moved towards the most distant flanking genes (*HACE1* and *LAMA4*, located 3.6 and 3.7 Mb, respectively, from *FOXO3*) in the 46-gene neighbourhood, as well as towards more proximally located flanking genes (*ATG5* and *AMD1*, located 2.1 and 2.3 Mb, respectively, from *FOXO3*), resulting in the formation of a tight transcription complex. *Bottom: FOXO3* mRNA expression was greater for cell lines containing the longevity-associated *G* allele of SNP *rs2802292* (mean ± SE; *P* < 0.001).

Amongst 110 single nucleotide polymorphisms (SNPs) within *FOXO3* and 5 kb of its flanking DNA 41 SNPs were found to be associated with longevity (living to ≥ 95 years of age) in American men of Japanese ancestry living on the Hawaiian island of Oahu, and who were part of the Kuakini Honolulu Heart Program. Nucleotide changes in 13 of these SNPs disrupted binding sites for 18 transcription factors having roles in growth, differentiation, stem cell maintenance, energy sensing and muscle homeostasis. In modelling studies involving the WashU Genome Browser those 13 SNPs were found to be connected to the *FOXO3* promoter via RNA II polymerase binding and likely formed a longevity-associated haplotype – or *cis*-regulatory unit. The functionality of these SNPs was confirmed by semi-quantitative PCR of *FOXO3* mRNA in genotypically different lymphoblastoid cell lines.

These new findings highlight the fact that genotype-phenotype correlations commonly reported in the study of complex traits often focus on single protein-coding genes but ignore gene neighborhoods. It has been suggested [3] that physical interactions between genes themselves might be an additional contributory factor in the omnigenic model proposed recently to explain the “missing heritability” evident from large-scale genome-wide association studies of complex polygenic traits [4]. Confirmation of this will require further research.

It would now appear that modulation of *FOXO3* activity could have an amplifier effect on genes in its neighborhood. This would complement the transcriptional effects that FoxO3 has on expression of a wide array of specific genes across the genome.

The well-known longevity-associated *TOMM40-APOE-APOC1* cluster of genes resides in a linkage disequilibrium block on chromosome 19q13.2 [5]. Could this complex be part of another neighbourhood of genes having roles in healthy aging?

Hopefully, the recent *FOXO3* findings will inspire others to look for additional longevity gene neighborhoods exhibiting gene-gene interactions (see review: [6]). These novel findings provide considerable food for thought in unravelling the intricate mechanisms responsible for longevity and other complex polygenic conditions.

## References

[1] Willcox BJ (2016). Aging Cell.

[2] Donlon TA (2017). Aging Cell.

[3] Morris BJ (2017). Circ Cardiovasc Genet.

[4] Boyle EA (2017). Cell.

[5] Bekris LM (2012). J Hum Genet.

[6] Elizondo LI (2009). Curr Genomics.

